# Transitions Between Home and Unscheduled Care in the Last Year of Life

**DOI:** 10.23889/ijpds.v9i5.2782

**Published:** 2024-09-18

**Authors:** Luciana Pedro, Richard Taylor, Sarah Bowers, Sarah Mills, Robin Alexander, Colin McCowan

**Affiliations:** 1 School of Medicine, University of St Andrews; 2 Yale New Haven Health System

Almost half of Scottish people who die currently die in hospital, which may represent a complex need or lack of alternative options. Nearly 95% of people use unscheduled care in their last year of life, which includes the Emergency Department (ED) – the outcome of ED attendance is often hospital admission. This increased use of unscheduled care services is associated with high costs and poor patient outcomes. This study analyses sequences of patient visits and describes patterns of ED attendance and inpatient admissions in the patients' final year of life to help understand their healthcare journeys.

This work examines the use of ED attendance and hospital admissions in NHS Tayside and NHS Fife between 2000 and 2021 for people in the last year of life. The retrospective cohort consists of all patients 18+ who died and visited the ED or were admitted to the hospital at least once. From a study population of 981,879, 164,506 (17%) people died, with 148,734 (90%) people having an ED visit or hospital admission. Data was divided for the 52 weeks before death, with three possible states for each week: home, ED visit, and admission.

A preliminary analysis shows high and significant variability in ED attendance and hospital admissions with distinct usage patterns. A better understanding of the patients' characteristics within each pattern may allow for potential interventions that improve patient care by reducing avoidable ED attendances or hospital admissions in the last year of life.

